# First detection of a lizard-associated papillomavirus in the splendid japalure (*Japalura splendida*) from southwestern China

**DOI:** 10.3389/fmicb.2025.1590538

**Published:** 2025-07-28

**Authors:** Zhige Tian, Tingjie Li, Yuping Fan, Jiayi Li, Sirong Luo, Wanxin Cao, Qing Pan, Xiaoliang Hu

**Affiliations:** ^1^Faculty of Agriculture, Forestry and Food Engineering, Yibin University, Yibin, China; ^2^College of Veterinary Medicine, Qingdao Agricultural University, Qingdao, China

**Keywords:** evolution, *Japalura splendida*, lizard, papillomavirus, reptile

## Abstract

**Introduction:**

Papillomaviruses have been previously identified in mammals, avians and fish. However, few numbers of reptiles’ PVs have been characterized.

**Methods:**

This study investigated the oral cavity of the splendid japalure (*Japalura splendida*) in southwestern China using high-throughput sequencing. The presence of papillomavirus strain JsPV in oral samples was confirmed using PCR with consensus primers.

**Results and discussion:**

In this study, a papillomavirus strain, designated JsPV, in the oral cavity of the splendid japalure (Japalura splendida) in southwestern China. The complete JsPV genome was sequenced, comprising  222 bp. Phylogenetic analysis based on the L1 protein revealed that JsPV clustered closely with gecko-derived strains (HfrePV1 and HfrePV2) and other sauropsid-associated papillomaviruses, while remaining distinct from mammalian- and fish-associated lineages. These findings provide insights into the evolutionary origins of papillomaviruses in reptiles.

## 1 Introduction

Papillomaviruses (PVs) have been detected across all vertebrate taxa, including reptiles, and constitute a diverse family of non-enveloped, double-stranded DNA viruses with genomes ranging from 7 to 8 kb in length. To date, only a limited number of non-mammalian PVs have been characterized, including yellow-necked francolin (*Francolinus leucoscepus*), common chaffinch (*Fringilla coelebs*), northern fulmar (*Fulmar glacialis*), African grey parrot (*Psittacus erithacus*), Adélie penguin (*Pygoscelis adeliae*), green sea turtle (*Chelonia mydas*), loggerhead sea turtle (*Caretta caretta*), gilt-head bream fish (*Sparus aurata*), and Asian house gecko (*Hemidactylus frenatus*) (Agius et al., 2019). Papillomaviridae genomes typically encode early regulatory proteins (E1 and E2) and late structural proteins (L1 and L2) (Agius et al., 2019). E1 serves as an adenosine triphosphate (ATP)-dependent DNA helicase, playing a key role in viral genome replication and episomal amplification, thought to be essential throughout the viral life cycle (Bergvall et al., 2013). To date, over 230 types of human PVs (HPVs) and 159 types of non-human PVs have been identified (Van Doorslaer et al., 2017). HPVs account for an estimated 27.9 to 30.0% of cancer cases globally (Zur Hausen, 2009; Bravo et al., 2010). PVs are implicated not only in human malignancies but also in neoplasms affecting various animal species. Notable examples include bovine papillomavirus, cottontail rabbit papillomavirus, rodent papillomavirus, feline papillomavirus, and canine oral papillomavirus, all of which have been recognized for their oncogenic potential in their respective hosts (Gil da Costa and Medeiros, 2014; Uberoi and Lambert, 2017; Medeiros-Fonseca et al., 2023). In reptiles, PVs are associated with mucocutaneous and cutaneous epithelial proliferative lesions (Herbst et al., 2009); however, no clear link has been established between PV infection and tumor development in these species.

The splendid japalure (*Japalura splendida*) is native to southwestern China, including Yunnan, Sichuan, Chongqing, and Hubei provinces, where it primarily inhabits forest edges (Huang et al., 2019). It is an insectivorous species and is commonly maintained in captivity as a pet (Tian et al., 2022). In this study, a papillomavirus was identified for the first time in the oral cavity of splendid japalure using high-throughput sequencing technology and virus-specific polymerase chain reaction (PCR) analysis. This discovery provides insights into the characteristics and evolutionary history of PVs in lizards.

## 2 Materials and methods

### 2.1 Sample preparation, DNA sequencing, and sequence analysis

Samples were obtained from a splendid japalure near the Jinsha River (28°64′12″N, 104°27′22″E) in the absence of overt clinical symptoms. Oral samples were collected using sterile swabs, which were placed in RNase-free tubes and transported on dry ice to Novogene Bioinformatics Technology Co., Ltd. (Beijing, China). High-throughput sequencing technology, including DNA sequencing and subsequent bioinformatic analyses, was performed as described previously (Liu et al., 2023). Briefly, DNA was extracted from oral samples using the E.Z.N.A.^®^ Stool DNA Kit (Omega BioTek, Norcross, GA, United States). A commercial library preparation kit [Rapid Plus DNA Lib Prep Kit for Illumina (RK20208)] was employed to construct the DNA library (ABclonal, Wuhan, China) according to the manufacturer’s protocol (Wang et al., 2024). Subsequently, library quality was assessed on the Agilent 5400 system (AATI) and quantified by real-time PCR (1.5 nM). The qualified libraries were pooled and sequenced on Illumina platforms with PE150 strategy in Novogene Bioinformatics Technology Co., Ltd. (Beijing, China), according to effective library concentration and the data amount required. Raw reads for clonal reads and low sequencing quality tails were processed using Trimmomatic (Bolger et al., 2014). Clean reads were assembled using the MEGAHIT software (Li et al., 2016) and were taxonomic classification using VIRify pipeline (Rangel-Pineros et al., 2023). All the contigs were split into high confidence (HC), low confidence (LC) and putative prophage (PP) sets. The assigned taxonomy was based on the informative ViPhOG hits per contig and performed on genus, family, subfamily (Moreno-Gallego and Reyes, 2021). The open reading frames (ORFs) in the viral genome were predicted by the BLASTx search results. The protein domains were identified and annotated using the NCBI conserved domain search (*E*-value <10^−5^) (Marchler-Bauer and Bryant, 2004).

### 2.2 Detection of papillomavirus in oral samples by PCR

Papillomavirus-specific primers for complete genome sequencing were designed based on known sequences (Supplementary Table S1). PCRs were conducted in a total of volume of 50 μL containing 5 μL of 10× buffer, 3 μL of dNTPs mixture (2.5 mM), 5 μL (10 ng) of DNA, 1 μL of forward primer (10 μM), 1 μL of reverse primer (10 μM), 0.5 μL (5 U) of LA Taq polymerase (TaKaRa, Tokyo, Japan), and 34.5 μL of sterile water. PCR was conducted with the cycling parameters: 94°C for 5 min, followed by 35 cycles at 94°C for 0.5 min, 56°C for 0.5 min, and 72°C for 2 min, and a final extension at 72°C for 10 min (Hu et al., 2015). Prior to sequencing, amplified products were cloned into the pMD18-T vector (TaKaRa). Three independent clones were sequenced using Sanger method with universal primers (M13F: AGGGTTTTCCCAGTCACG; M13R: CAGGAAACAGCTATGAC) to confirm sequence integrity. Complete sequences were manually assembled and alignment using Vector 10, and DNASTAR, respectively.

### 2.3 Phylogenetic and homology model analyses

The amino acid sequence of L1 protein was aligned with Pairwise nucleotide sequence similarity using Needleman–Wunsch algorithm of global alignment.^2^ Phylogenetic trees were constructed based on L1 protein sequences using the maximum-likelihood (ML) method with the LG + I + G + F model in MEGA v7.0. Bootstrap values were estimated for 1,000 replicates. Homology modeling of papillomavirus proteins was conducted using Phyre2.2 (PHYRE2 Protein Fold Recognition Server, ic.ac.uk).

## 3 Results and discussion

**Figure 1:**
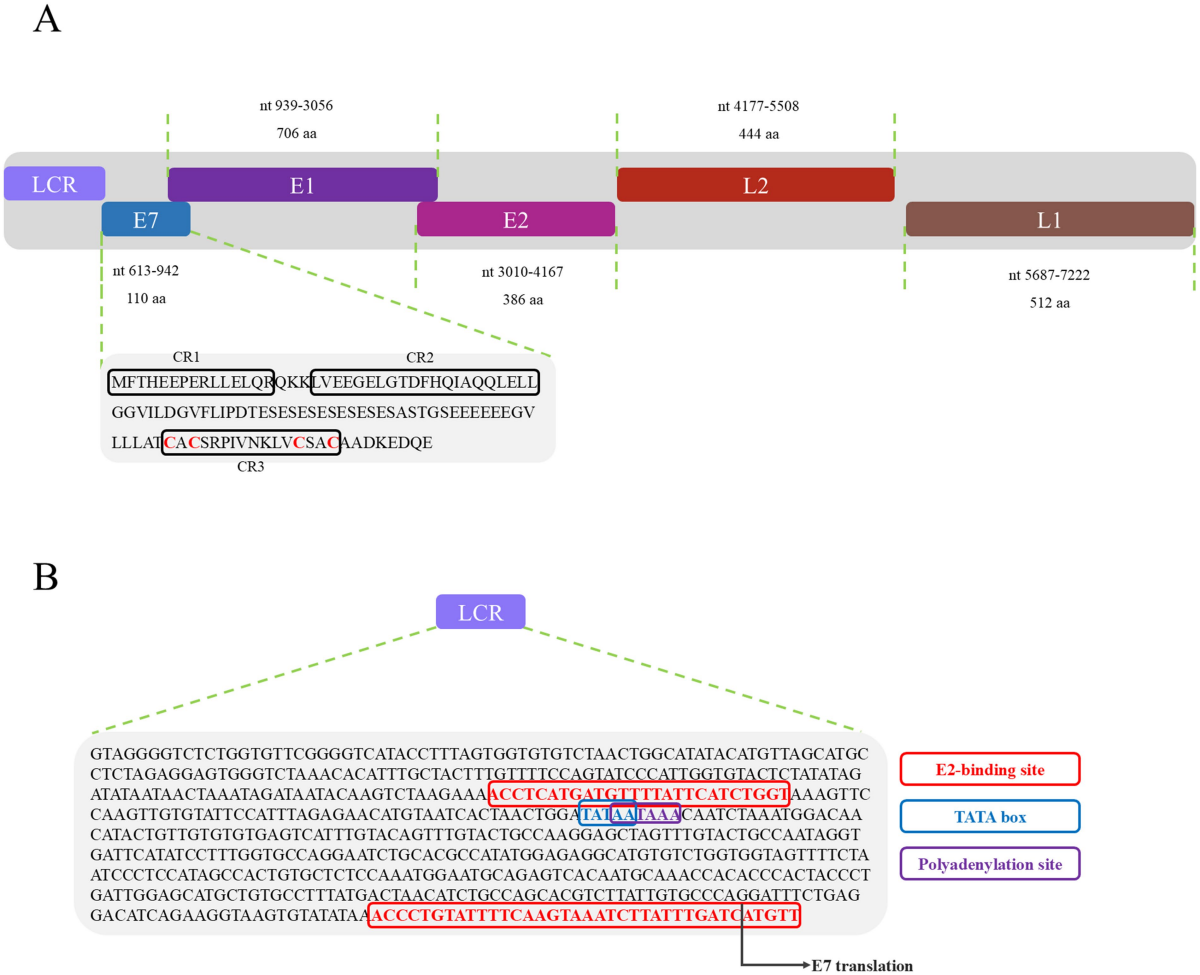
Genomic organization of JsPV. **(A)** Illustration of the genomic organization of JsPV, including LCR, E7, E1, E2, L2, and L1. The sequence and motif annotation of E7 protein, black boxes indicate conserved regions referred to CR1, CR2, and CR3, and red letters indicate putative Zn-binding domain. **(B)** Representation of the LCR motifs identified in JsPV. Coloured boxes represent different regulatory elements as per key provided, including E2-binding site, TATA box, polyadenylation site.

**Figure 2:**
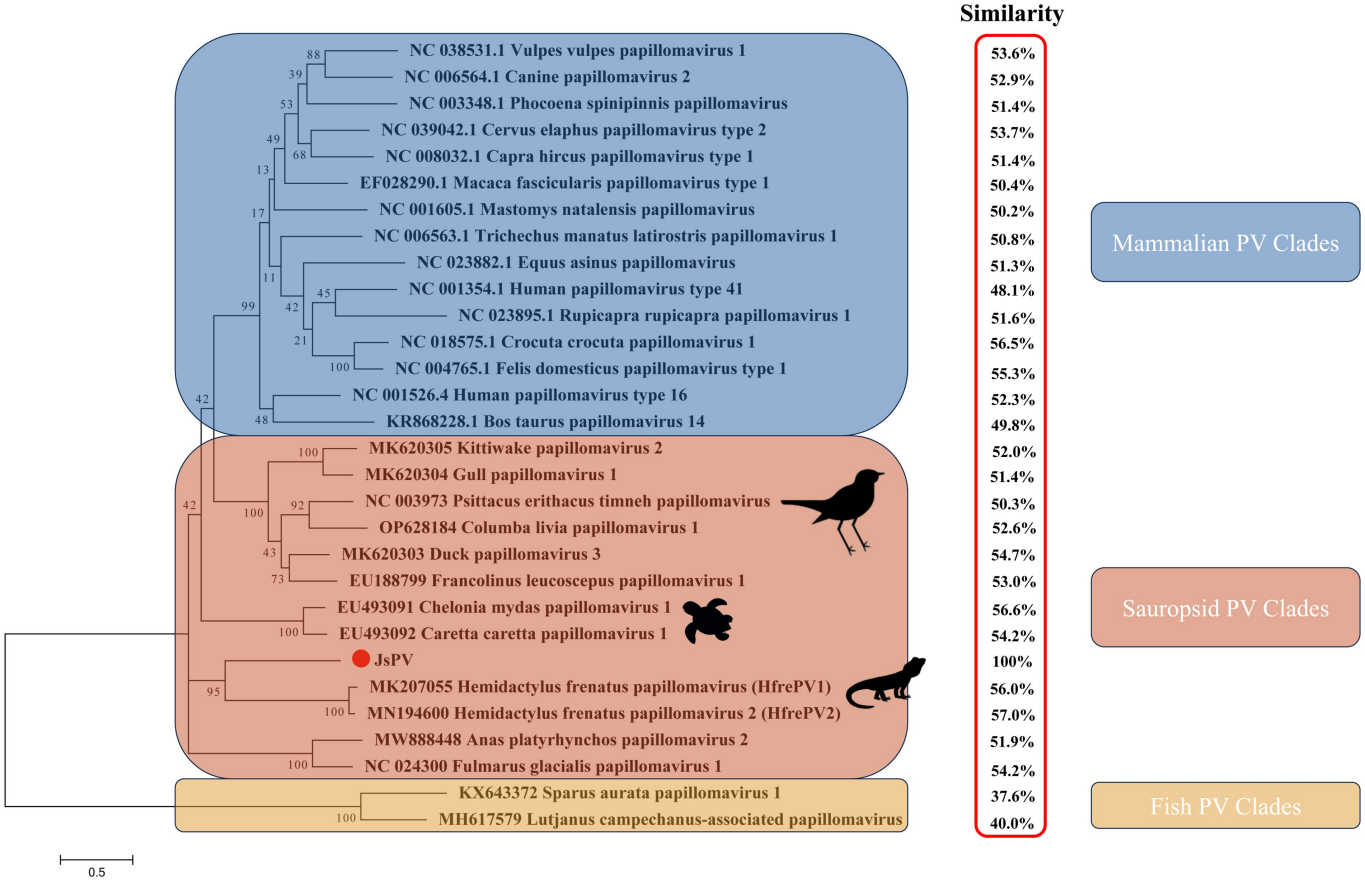
Maximum likelihood phylogenetic tree showing the relationship between JsPV and other papillomaviruses based on the L1 protein. The results of the L1 protein similarity comparison are showed in the red box.

PVs are widely distributed across vertebrate and invertebrate species, including mammals (Varga et al., 2014; Bravo and Félez-Sánchez, 2015; Brücher and Jamall, 2014; Herbster et al., 2012), birds (Terai et al., 2002; Tachezy et al., 2002; Varsani et al., 2014; Gaynor et al., 2015; Varsani et al., 2015), fish (López-Bueno et al., 2016), and reptiles (Agius et al., 2019; Herbst et al., 2009; Lange et al., 2011). Among reptiles, previous studies have reported papillomavirus infections only in turtles and geckos, with viral strains distinct from those identified in other hosts (Agius et al., 2019; Herbst et al., 2009). In this study, a papillomavirus was identified for the first time in the oral cavity of a splendid japalure in the absence of visible lesions. The complete genome of this strain, designated JsPV, was amplified by PCR, revealing a genome length of 7,222 bp with a GC content of 43%. Conserved early proteins E1 (706 amino acids) and E2 (386 amino acids), along with late structural proteins L1 (512 amino acids) and L2 (444 amino acids), were identified (Figure 1A). A 612 bp region (8.5% of the genome) located between the stop codon of L1 and the start of E7 was identified, corresponding to the long control region (LCR) observed in other PVs (Figure 1B). It contained a polyadenylation (PolyA) site [257–262 nucleotides (nt)], a TATA box (254–258 nt) within an AT-rich region, one E2-binding site (ACC-N_20_-GGT) located at 177–202 nt and two modified putative E2-binding sites (AAC-N_7-32_-GGT) were found at 177–189 nt and 581–618 nt. No Nf1 or Sp1 binding sites matching the motifs identified in JsPV were detected. The putative E7 oncoprotein consisted of 110 amino acids (nucleotides (nt) 613–942), with a zinc-binding motif (CxxC) located in the C-terminal region (84–99 amino acids) and non-folded CR1 and CR2 motifs, although it lacked the conserved retinoblastoma protein (pRb) binding motif (L-x-C-x-E) (Figure 1A). Structural analysis indicated that the E7 protein of JsPV shared similarity with those of gecko-derived PVs (Supplementary Figure S1). Multiple sequence alignment indicated that L1 protein of JsPV shared 37.6–57% identity with mammalian- and sauropsid-associated PVs (Figure 2). Compared to the genomic architecture of other known sauropsid PVs, JsPV, along with HfrePV1 and HfrePV2, encoded only five proteins (E7, E1, E2, L2, and L1), lacking the E6 gene—the fewest reported among lacertilian PVs. E6 is a small oncoprotein (Boulet et al., 2007) that can form a trimeric complex with the E6AP ubiquitin ligase and p53 (Tan et al., 2012), leading to cell transformation (Liu et al., 2002) and immortalization (Boon et al., 2015), suggesting that lacertilian PVs may lack the molecular capacity to induce tumorigenesis. Previous studies have identified PVs in the genera *Hemidactylus*, *Gehyra*, and *Lepidodactylus* in Australia (Agius et al., 2019). However, the present study provides the first evidence of PVs in the genus *Japalura*, suggesting that these viruses may have originated from a distinct ancestral lineage in lizards and may have adapted to specific ecological niches within sympatric Lacertidae. Furthermore, the splendid japalure is endemic to China and inhabits faunal assemblages distinct from those of gecko-derived PVs. This finding suggests that there is a potential pathogenic risk of papillomavirus transmission to other reptiles. Further investigations are needed to explore the broader distribution of these viruses across China.

Phylogenetic analysis demonstrated that the lizard-derived JsPV clustered closely with gecko-associated strains (HfrePV1 and HfrePV2), forming a distinct clade separate from those associated with snakes, birds, turtles, and mammals (Figure 2). This finding suggests that species from geographically distinct regions may have originated from ancestral lizards and their associated PVs. Additionally, turtle-associated PVs formed a unique cluster within the sauropsid clade, supporting the hypothesis that PVs in reptiles have evolved independently with limited interspecific host transmission.

Overall, this study provides evidence of PV infection in lizards without obvious clinical symptom, the same as avian papillomaviruses. Whether the JsPV could develop a persist infection or a simply present in the oral cavity was unknown in lizard. Future research should focus on elucidating the evolutionary origins of reptilian PVs and expanding our current understanding of sauropsid viral genomes.

## Data Availability

The data presented in the study are deposited in the online repository. The names of the repository/repositories and accession number(s) can be found below: NCBI–PRJNA1233463, SRR32623559, PV259886.

## Footnotes

^2^ https://www.ebi.ac.uk/jdispatcher/psa

## Supplementary material

SUPPLEMENTARY FIGURE S1 Structural models of putative E7 ORFs of HPV-45 (A), CcPV1 (B), HfrePV1 (C), and JsPV (D). Homology models for JsPV based on the experimentally derived sequence for HPV45 E7.
