# Supplementary figures and images for: First detection of a lizard-associated papillomavirus in the splendid japalure (Japalura splendida) from southwestern China
Source: Front Microbiol. 2025 Jul 28;16:1590538. doi: 10.3389/fmicb.2025.1590538 (PMC12336156; doi:10.3389/fmicb.2025.1590538)

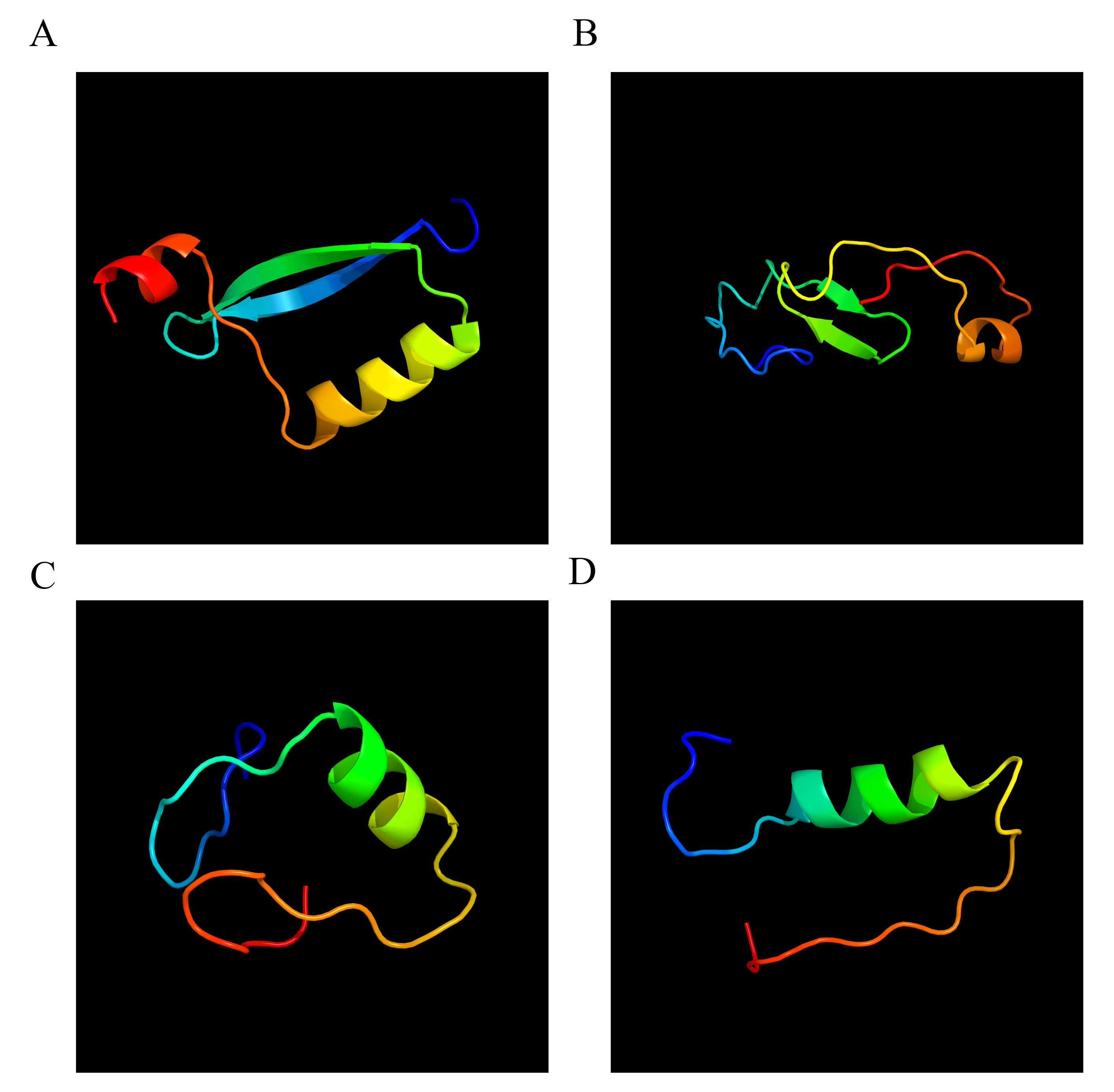

Supplement: SUPPLEMENTARY FIGURE S1 — Structural models of putative E7 ORFs of HPV-45 (A), CcPV1 (B), HfrePV1 (C), and JsPV (D). Homology models for JsPV based on the experimentally derived sequence for HPV45 E7. [file Image_1.tif]
