# Supplementary material for: First detection of a lizard-associated papillomavirus in the splendid japalure (Japalura splendida) from southwestern China
Source: Front Microbiol. 2025 Jul 28;16:1590538. doi: 10.3389/fmicb.2025.1590538 (PMC12336156; doi:10.3389/fmicb.2025.1590538)
Supplement: Supplementary file 2 [file Table_1.docx]

Table S1 Primers used for ampliﬁcation of JsPV sequences

| Name | Sequence | Position |
| --- | --- | --- |
| 1-1F | GTAGGGGTCTCTGGTGTTCG | 1–20 |
| 1-1R | TGTACTGGCTCATTCTTGGTC | 931–951 |
| 2-1F | AGGGAGTACTTCTGCTTGCA | 845–864 |
| 2-2R | TCTTTATATGAGTCCTTGGA | 1 710–1 729 |
| 3-3F | GGCCAAGAAGAACATCCTGA | 1 595–1 615 |
| 3-3R | CCCATTATGCACATACAGCA | 2 495–2 515 |
| 4-4F | TACATCACAGTTAAGGTATGC | 2 270–2 290 |
| 4-4R | ACTTTATGTTATGTTCAATCA | 3 245–3 265 |
| 5-5F | GGATTAGAAGACATAGAGGACAA | 2 982–3 004 |
| 5-5R | CTATATGGCAGCTTTATACTC | 5 001–5 021 |
| 6-6F | AGCATTGCCTCTACATCATT | 4 885–4 904 |
| 6-6R | ATTCTTCTTCACTGATATGT | 6 744–6 763 |
| 7-7F | TATTGGATCACTCATATCAA | 6 647–6 666 |
| 7-7R | TCACTTTCTCCTTTTTTTCA | 7 203–7 222 |
